# Human CD8+ T cells mediate protective immunity induced by a human malaria vaccine in human immune system mice

**DOI:** 10.1016/j.vaccine.2016.08.006

**Published:** 2016-08-31

**Authors:** Xiangming Li, Jing Huang, Min Zhang, Ryota Funakoshi, Dutta Sheetij, Roberta Spaccapelo, Andrea Crisanti, Victor Nussenzweig, Ruth S. Nussenzweig, Moriya Tsuji

**Affiliations:** a HIV and Malaria Vaccine Program, Aaron Diamond AIDS Research Center, Affiliate of The Rockefeller University, 455 First Avenue, New York, NY 10016, United States; b Department of Pathology, New York University School of Medicine, 550 First Avenue, New York, NY 10016, United States; c Malaria Vaccine Development Branch, Walter Reed Army Institute of Research, Silver Spring, MD, United States; d Università degli Studi di Perugia, Perugia, Italy; e Imperial College London, London, UK; f Division of Parasitology, Department of Microbiology, New York University School of Medicine, New York, NY, United States

**Keywords:** Ad, adenovirus, AAV9, adeno-associated virus serotype 9, CSP, circumsporozoite protein, FL, full-length, HIS, human immune system, Pf, *Plasmodium falciparum*, Py, *Plasmodium yoelii*, TCR, T-cell receptor, Human CD8+ T cell, Human immune system mice, Protection, Malaria, Recombinant adenovirus

## Abstract

A number of studies have shown that CD8+ T cells mediate protective anti-malaria immunity in a mouse model. However, whether human CD8+ T cells play a role in protection against malaria remains unknown. We recently established human immune system (HIS) mice harboring functional human CD8+ T cells (HIS-CD8 mice) by transduction with HLA-A∗0201 and certain human cytokines using recombinant adeno-associated virus-based gene transfer technologies. These HIS-CD8 mice mount a potent, antigen-specific HLA-A∗0201-restricted human CD8+ T-cell response upon immunization with a recombinant adenovirus expressing a human malaria antigen, the *Plasmodium falciparum* circumsporozoite protein (PfCSP), termed AdPfCSP. In the present study, we challenged AdPfCSP-immunized HIS-CD8 mice with transgenic *Plasmodium berghei* sporozoites expressing full-length PfCSP and found that AdPfCSP-immunized (but not naïve) mice were protected against subsequent malaria challenge. The level of the HLA-A∗0201-restricted, PfCSP-specific human CD8+ T-cell response was closely correlated with the level of malaria protection. Furthermore, depletion of human CD8+ T cells from AdPfCSP-immunized HIS-CD8 mice almost completely abolished the anti-malaria immune response. Taken together, our data show that human CD8+ T cells mediate protective anti-malaria immunity *in vivo*.

## 1 Introduction

Malaria is a mosquito-borne infectious disease caused by parasitic protozoans of the genus *Plasmodium.* Although medications and mosquito control efforts have limited the disease, malaria is still pandemic, with 198 million cases occurring in 2013, resulting in 584,000 fatalities [1]. These data underscore the need for new methods to control this disease, including more effective vaccines.

Most vaccine efforts are directed against the pre-erythrocytic stages [sporozoites (Spz) and liver stages], and blood stages [2]. The finding that vaccination with radiation-attenuated sporozoites (IrSpz) can induce complete protection (i.e., sterile immunity) against malaria infection not only in experimental animals but also in man [3], [4], [5], [6], [7] demonstrated the feasibility of effective vaccination against this disease. A number of mouse studies to date using *Plasmodium yoelii* and *Plasmodium berghei* parasites for challenge have shown that protective immunity against pre-erythrocytic stages is mediated in part by T cells, particularly CD8+ T cells. Firstly, the major role for CD8+ T cells was shown by studies in which *in vivo* depletion of CD8+ T cells abrogated Spz-induced protective immunity in mice [8], [9]. Secondly, the adoptive transfer of CD8+ T-cell clones specific for the immunodominant CD8+ T-cell epitope of the *P. berghei* or *P. yoelii* circumsporozoite protein (CSP), a major Spz antigen, confers protection against Spz challenge in naïve mice [10], [11], [12]. Using transgenic mice expressing a T-cell receptor (TCR), based on the TCR sequence of CD8+ T cells recognizing a CD8+ T-cell epitope present in *P. yoelii* CSP (PyCSP), transgenic CD8+ T cells were shown to mediate protection against malaria [13]. Finally, a single immunizing dose of a recombinant adenovirus expressing the PyCSP, AdPyCSP, has been shown to induce a potent protective anti-malarial immunity, which was mediated primarily by CD8+ T cells [14].

Beyond mouse model, Hoffman’s group has recently shown that intravenous (IV) immunization of IrSpz of *Plasmodium falciparum*, PfSPZ vaccine, is very effective in inducing a high frequency of malaria-specific CD8+ T cells in the liver of nonhuman primates [15]. More recently the same group showed that immunization of multiple doses of their IrPfSPZ vaccine by IV induced a high level of PfSPZ-specific T-cell responses, including that of CD8+ T cells, and conferred protection in six out of six (100%) human vaccinees against malaria challenge [16]. Another recent study has shown that the administration of live *P. falciparum* Spz by bites of infected mosquitoes, followed by chloroquine treatment induced significant malaria-specific pluripotent effector memory T-cell responses in vaccinated volunteers and protected all of them (10 out of 10) upon malaria challenge [17]. With regards to human malaria vaccines based on viral vectors, a small number have entered human clinical trials in recent years. In a phase I clinical trial, 15 volunteers were primed with plasmid DNA encoding *P. falciparum* CSP (PfCSP) and apical membrane antigen-1 and then boosted with human adenovirus serotype 5 (Ad5) expressing the same antigens. This DNA priming/adenovirus boost immunization regimen induced sterile protection in four (27%) vaccinated subjects [18]. In a phase IIa clinical trial, vaccination using a priming-boost regimen based on chimpanzee adenovirus and modified Ankara vaccinia virus, both expressing *P. falciparum* thrombospondin adhesive protein fused to multiple epitopes derived from several malaria antigens, induced sterile protection in 21% (3 out of 14) of subjects and delayed patency in 36% (5 out of 14) of subjects [19]. Although the degree of protection in these trials was modest, both trials exhibited a trend toward a higher-level vaccine-induced CD8+ T-cell response in protected individuals [18], [19]. Finally, it has been shown that the PfCSP also contains CD8+ T cell epitopes [20], [21] and can elicit a potent CD8+ T-cell response in humans upon immunization with various human malaria vaccines, including PfSPZ and AdPfCSP [22], [23], [24], [25], [26].

In a previous study, we established a human immune system (HIS) mouse model by transducing genes encoding human HLA-A∗0201 and human cytokines using a recombinant adeno-associated virus serotype 9 (AAV9) vector [27]. These mice express functional HLA-A∗0201-restricted human CD8+ T cells, and were therefore designated HIS-CD8 mice. In the present study, the role of vaccine-induced human CD8+ T cells in mediating protective immunity against malaria was investigated in HIS-CD8 mice.

## 2 Materials and methods

### 2.1 Ethics statement

All animal experiments were carried out in strict accordance with the Policy on Humane Care and Use of Laboratory Animals of the United States Public Health Service. The protocol was approved by the Institutional Animal Care and Use Committee (IACUC) at The Rockefeller University. Mice were euthanized using CO_2_, and every effort was made to minimize suffering. Human fetal liver samples were obtained via a non-profit partner (Advanced Bioscience Resources, Alameda, CA). As no information was obtained that would identify the subjects from whom the samples were derived, Institutional Review Board approval for their use was not required, as previously described [27].

### 2.2 Mice

NOD.Cg*^tm1Unc^ Prkdc^scid^ Il2rg^tm1Wjl^*/SzJ (NSG) mice exhibiting features of both severe combined immunodeficiency mutations and interleukin (IL)-2 receptor gamma-chain deficiency were purchased from Jackson Laboratories (Bar Harbor, ME) and maintained under specific pathogen-free conditions in the animal facilities at The Rockefeller University Comparative Bioscience Center.

### 2.3 Generation of HIS-CD8 mice

Recombinant AAV9 (rAAV9) vectors encoding human IL-3, IL-15, GM-CSF, and HLA-A∗0201 were constructed as previously described [27]. Four-week-old NSG mice were transduced with rAAV9 encoding HLA-A∗0201 by intrathoracic (IT) injection and with rAAV9 encoding HLA-A∗0201 and AAV9 encoding human IL-3, IL-15, and GM-CSF, by intravenous (IV) injection, as previously described [27]. Two weeks later, mice were subjected to 150-Gy total body sub-lethal irradiation for myeloablation, and several hours later, each transduced, irradiated mouse was engrafted intravenously with 1 × 10^5^ HLA-A∗0201+ matched, CD34+ human hematopoietic stem cells (HSCs). CD34+ HSCs among lymphocytes derived from HLA-A∗0201+ fetal liver samples were isolated using a Human CD34 Positive Selection kit (STEMCELL TECHNOLOGIES Inc. Vancouver, BC, Canada) [28]. At 14 weeks after HSC engraftment, the reconstitution status of human CD45+ cells in the blood of HIS-CD8 mice was determined by flow cytometric analysis, as previously described [27].

### 2.4 Recombinant adenovirus and transgenic parasites

A recombinant adenovirus serotype 5 (Ad5) expressing a green fluorescent protein (GFP) alone in its transgene, AdGFP, was previously constructed [29]. A recombinant Ad5 expressing *P. falciparum* CSP (AdPfCSP) was also previously constructed as described [29]. Briefly, a gene encoding a full length PfCSP was codon-optimized and synthesized, followed by being inserted into pShuttle-CMV, which was then used to make the recombinant AdPfCSP. HIS-CD8 mice were immunized with 5 × 10^10^ AdPfCSP virus particles [27], [29]. A transgenic *P. berghei* sporozoite expressing full-length *P. falciparum* CSP (FLPfCSP/Pb sporozoite) was generated as previously described [30], [31] and maintained at the Insectary Core Facility of New York University.

### 2.5 Intra-cellular cytokine staining (ICS)

Spleens were harvested from HIS-CD8 mice 10 days after vaccination with AdPfCS or AdGFP or from naïve HIS-CD8 mice. After isolation of lymphocytes, the cells were counted and used for ICS upon stimulation with synthetic peptides corresponding to the A2-restricted CD8+ T-cell epitopes of the PfCSP (YLNKIQNSL, KLRKPKHKKL and SLKKNSRSL) [20], [21]. Briefly, Lymphocytes were stimulated for 4–6 h using a pool of the synthetic peptides listed above or none (as a negative control) in the presence of brefeldin at 37 °C with 5% CO_2_. ICS assays were performed as previously described [16]. Briefly, after blocking with the anti-mouse CD16/CD32 antibody, lymphocytes were stained for surface markers with antibodies against CD45, CD3, and CD8. Next, lymphocytes were permeabilized with perm/wash solution (BD Biosciences, San Jose, CA), stained with the FITC-labeled anti-human IFN-γ antibody, fixed with 1% paraformaldehyde, and analyzed using a BD LSR II (BD Biosciences).

### 2.6 Staining with HLA-A∗0201 tetramer loaded with YLNKIQNSL peptide

An Allophyocyanin-labeled human HLA-A∗0201 tetramer loaded with the peptide YLNKIQNSL, corresponding to the PfCSP CD8+ T-cell epitope [20], [21], was provided by the NIH Tetramer Core Facility. HIS-CD8 mice were immunized with AdPfCSP, and 10 days later, the mice were challenged with 2 × 10^4^ live FLPfCSP/Pb sporozoites by IV injection. After 42 h, the spleens were harvested, and splenocytes were stained with APC-labeled human HLA-A∗0201 tetramer loaded with YLNKIQNSL and PE-labeled anti-human CD8 antibody (BioLegend, San Diego, CA). The percentage of HLA-A∗0201-restricted, PfCSP-specific CD8+ T cells among the total human CD8+ T-cell population was determined using a BD LSR II flow cytometer (Franklin Lakes, NJ).

### 2.7 Sporozoite challenge and assessment of parasite burden in the liver

FL-PfCSP/Pb sporozoites were obtained from salivary glands dissected from infected *Anopheles stephensi* mosquitoes 2 weeks after an infective blood meal. Sporozoite challenge experiments were carried out as described previously [30], [31]. Briefly, immunized mice were injected with 2 × 10^4^ live sporozoites via the tail vain, and 42 h after the challenge, the parasite burden in the liver was determined by measuring parasite-specific ribosomal RNA using a 7300 Real-Time PCR system (Applied Biosystems, Waltham, MA). Parasite burden was defined as the ratio of the absolute copy number of parasite ribosomal RNA to that of mouse *GAPDH* mRNA, and the percentage was calculated in relation to the parasite burden in naïve mice.

### 2.8 *In vivo* depletion of human CD8+ T cells

For the depletion of human CD8+ T cells, 0.1 mg of the rhesus IgG1 anti-human CD8 mAb MT807R1 [32], as well as rhesus recombinant IgG1 antibody, as an isotype control (both purchased from the NIH Nonhuman Primate Reagent Resource, Boston, MA), were administered to each HIS-CD8 mouse by intraperitoneal (IP) injection at 5 days and 2 days before sporozoite challenge. The spleen was harvested from each mouse 42 h after the challenge, and splenocytes were stained with the following antibodies (all purchased from BioLegend): Pacific Blue-labeled anti-human CD45, PerCP/Cy5.5-labeled anti-mouse CD45, PE-Cy7-labeled anti-human CD3, Alexa Fluor 700-labeled anti-human CD8, APC-Cy7-labeled anti-human CD4, and PE-labeled anti-human CD19. The degree of human CD8+ T-cell depletion was assessed using a BD LSR II.

### 2.9 Human IgM ELISA

The serum level of human IgM was determined by ELISA (Bethyl Laboratories, Montgomery, TX), as we previously performed [33].

### 2.10 Statistical analysis

All of the statistical analyses were done using GraphPad Prism (ver. 4.03) (GraphPad Software, Inc.). Bars in each figure represent geometric means. In all experiments, the values were log-transformed and then one-way ANOVA followed by a Dunnett’s test was employed to determine the differences between the groups. For a correlation analysis, linear regression analysis was applied to % of PfCSP-specific CD8+ T cells among the total human CD8+ T-cell population and % of relative parasite burden in liver, as 100% being the parasite burden in naïve and challenged mice.

## 3 Results

### 3.1 Establishment of HIS-CD8 mice

**Fig. 1:**
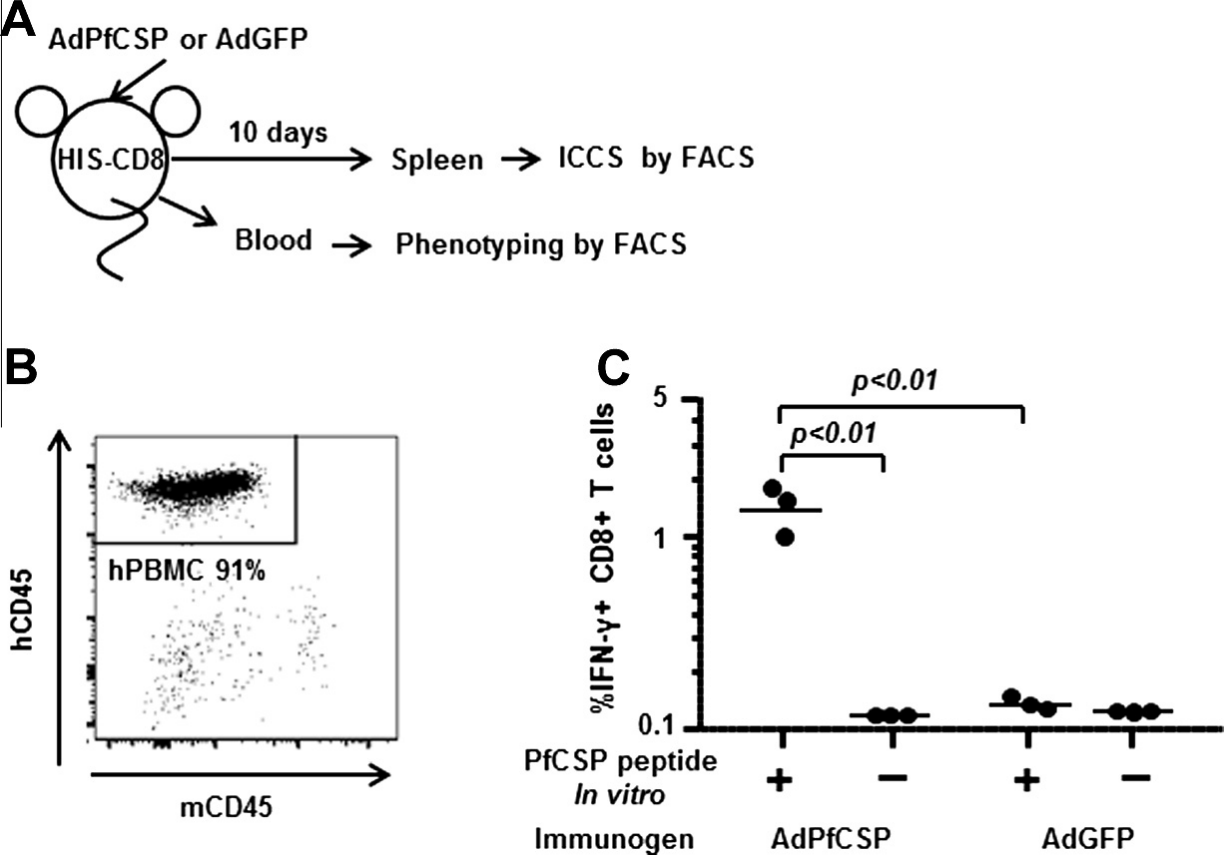
AdPfCSP vaccination induced PfCSP-specific human CD8+ T cells secreting human IFN-γ in HIS-CD8 mice. (A) A group of three HIS-CD8 mice were immunized by IM injection with AdPfCSP or AdGFP at 10^10^ virus particles/mouse, and 10 days later, spleens were harvested from the both groups and lymphocytes isolated. (B) Prior to immunization, blood was collected from HIS-CD8 mice, and the percentage of human CD45+ cells in the blood was determined by a flow cytometric analysis. (C) Using lymphocytes isolated from the spleen, ICS was performed to assess the percentage of PfCSP-specific human CD8+ T cells secreting human IFN-γ among total CD8+ T cells by a flow cytometric analysis.

As described previously [27], we reconstituted functional human CD8+ T cells by delivering genes encoding HLA-A∗0201 and then selected human cytokines using rAAV9 as a vector. Briefly, NSG mice were administered rAAV9 encoding HLA-A∗0201 by both IV and IT injection plus an IV injection of a cocktail of rAAV9 vectors encoding human IL-3, IL-15, and GM-CSF, followed by engraftment of HLA-A∗0201+ CD34+ HSCs. Determination of the phenotypes of peripheral blood mononuclear cells (PBMCs) by flow cytometric analysis after 14 weeks revealed that human CD45+ cells constituted more than 90% of the total PBMC population in the HIS mice (Fig.1B). These HIS-CD8 mice can mount human IgM in their sera (data not shown).

### 3.2 Correlation of the level of the PfCSP-specific CD8+ T-cell response induced in HIS-CD8 mice and the level of malaria protection

**Fig. 2:**
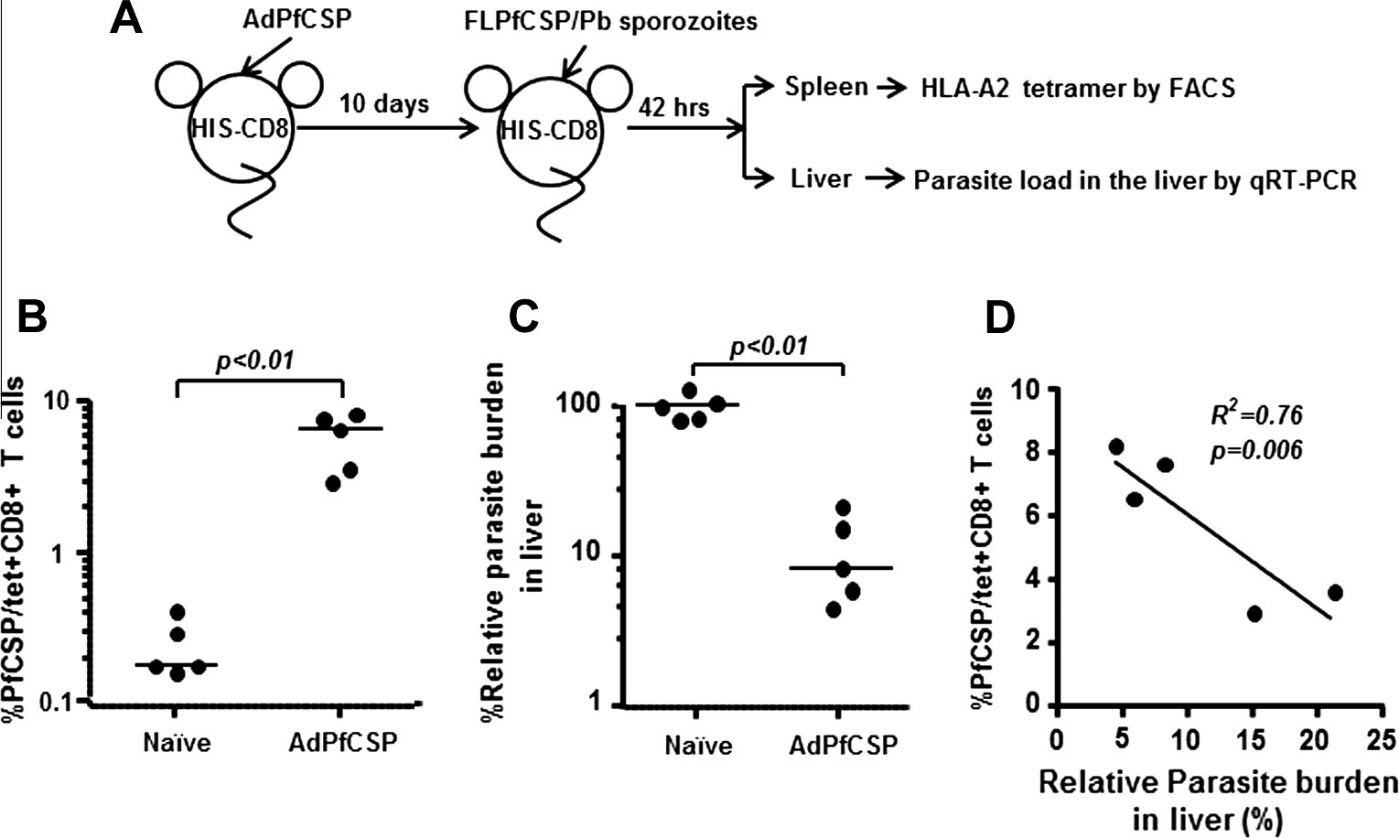
PfCSP-specific CD8+ T-cell response and protective anti-malaria response induced in HIS-CD8 mice. (A) Five HIS-CD8 mice were immunized by i.m. injection with AdPfCSP at 5 × 10^10^ virus particles/mouse. After 10 days, the AdPfCSP-immunized mice, as well as five naïve HIS-CD8 mice, were challenged by i.v. injection with 2 × 10^4^ live FLPfCSP/Pb sporozoites. (B) After 42 h, both groups of HIS-CD8 mice were sacrificed, and the percentage of HLA-A2-restricted, PfCSP-specific CD8+ T cells among total CD8+ T cells in the spleen was determined using HLA-A2 tetramer staining and flow cytometric analysis. (C) The parasite load in the liver was determined by qRT-PCR analysis. (D) The correlation between the percentage of PfCSP-specific CD8+ T cells and the relative parasite load in the liver was assessed using Prim GraphPad 5.0 software.

A group of three HIS-CD8 mice were first immunized with 5 × 10^10^ virus particles of AdPfCSP or AdGFP, as a control, by intramuscular (IM) injection (Fig.1A). IM route was chosen because of the practicality and also the safety issue. Ten days later, spleens were removed from a group of immunized HIS-CD8 mice, and ICS was performed in the presence or absence of a pool of PfCSP-derived CD8+ T-cell epitopes to determine the percentage of PfCSP-specific human CD8+ T cells secreting human IFN-γ. We found that AdPfCSP-immunized, but not AdGFP-immunized, HIS-CD8 mice were able to induce a significant level of PfCSP-specific human CD8+ T-cell response (Fig.1C). In the next experiments, another group of five AdPfCSP-immunized, as well as five naïve HIS-CD8 mice, were challenged IV with 2 × 10^4^ live FLPfCSP/Pb sporozoites 10 days after immunization (Fig.2A). The level of the PfCSP-specific human CD8+ T-cell response induced in the spleen was determined 42 h after the sporozoite challenge by flow cytometric analysis using an HLA-A∗0201 tetramer loaded with YLNKIQNSL, a peptide representing the HLA-A∗0201-restricted human CD8+ T-cell epitope present in PfCSP [20], [21]. We found that a PfCSP-specific, HLA-A∗0201-restricted human CD8+ T-cell response was generated only after vaccination with AdPfCSP followed by challenge with live FLPfCSP/Pb sporozoites and not after challenge with FLPfCSP/Pb sporozoites alone (Fig.2B). When we determined the parasite load in the liver of both AdPfCSP-immunized and naïve HIS-CD8 mice at 42 h after FLPfCSP/Pb sporozoite challenge, we found that the parasite load was reduced by 91% in AdPfCSP-immunized mice compared with the naïve control group, indicating that immunization with AdPfCSP conferred protection against malaria (Fig.2C). Importantly, a significant correlation (*p* = 0.006) was observed between the level of the PfCSP-specific human CD8+ T-cell response and the level of malaria protection (Fig.2D).

### 3.3 Abolishment of malaria protection observed in AdPfCSP-immunized HIS-CD8 mice by *in vivo* depletion of human CD8+ T cells

**Fig. 3:**
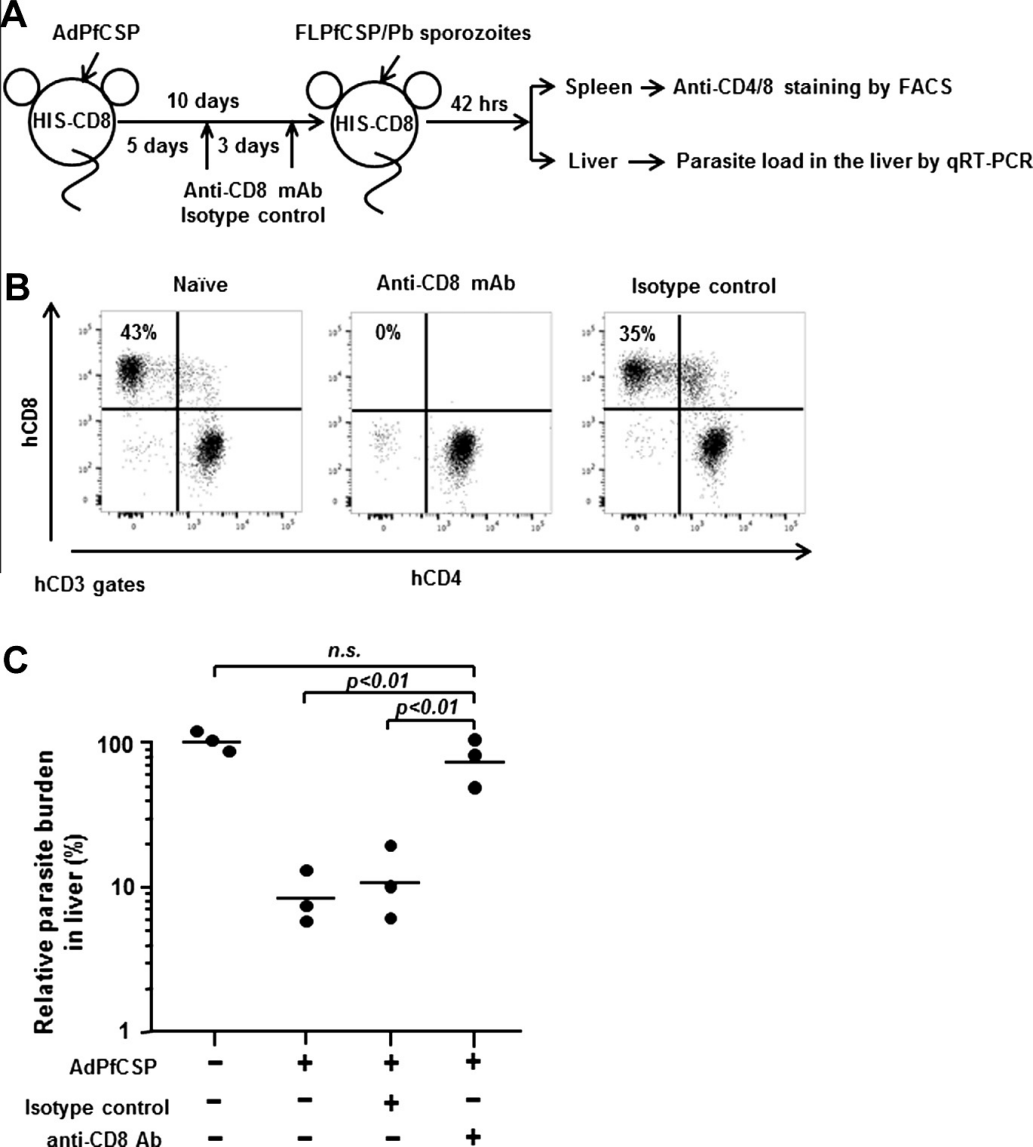
*In vivo* depletion of human CD8+ T cells abolished protective anti-malaria immunity induced in AdPfCSP-immunized HIS-CD8 mice. (A) Six HIS-CD8 mice were immunized by IM injection with AdPfCSP at 5 × 10^10^ virus particles/mouse. A group of three AdPfCSP-immunized HIS-CD8 mice received two 0.1 mg/mouse doses of anti-CD8 mAb, MT807R1, or rhesus recombinant IgG1, as an isotype control, by IP injection on days 5 and 8 post-AdPfCSP immunization. After 10 days, three AdPfCSP-immunized HIS-CD8 mice, three AdPfCSP-immunized/human CD8+ T-cell depleted HIS-CD8 mice, and three naïve HIS-CD8 mice were challenged by IV injection with 2 × 10^4^ live FLPfCSP/Pb sporozoites. (B) After 42 h, both groups of HIS-CD8 mice were sacrificed, and the percentages of human CD8+ and CD4+ T cells among the total human T-cell population were determined by flow cytometric analysis. (C) The parasite load in the liver was determined by qRT-PCR.

In order to determine the role of human CD8+ T cells in mediating the protective anti-malaria immune response observed in HIS-CD8 mice, a group of AdPfCSP-immunized HIS-CD8 mice received two IP injections of the monoclonal anti-human CD8+ T-cell antibody, MT807R1 (0.1 mg/mouse/injection) [32], 5 days and 8 days after AdPfCSP immunization (Fig.3A). Rhesus IgG1 antibodies were administered to another group of AdPfCSP-immunized HIS-CD8 mice, as an isotype control. At 10 days after AdPfCSP immunization, naïve HIS-CD8 mice, AdPfCSP-immunized HIS-CD8 mice, and AdPfCSP-immunized, anti-human CD8+ antibody-treated HIS-CD8 mice were challenged with live FLPfCSP/Pb sporozoites. The spleen and liver were harvested from each mouse 42 h later to determine the percentage of human CD8+ T cells and the parasite burden, respectively (Fig.2A). Flow cytometric analysis confirmed that administration of anti-human CD8+ antibody, but not the isotype control antibody, resulted in 100% depletion of human CD8+ T cells in the spleen of HIS-CD8 mice (Fig.3B). Most importantly, we found that the *in vivo* depletion of human CD8+ T cells almost completely abolished the inhibition of live-stage malaria parasites observed in HIS-CD8 mice upon AdPfCSP immunization (Fig.3C). These results indicate that human CD8+ T cells mediate the protective anti-malaria immune response induced in HIS mice by the human malaria vaccine examined in this study.

## 4 Discussion

The role of CD8+ T cells in protective immunity against malaria, particularly immunity against the pre-erythrocytic stages of the parasite, has been examined in a number of mouse studies using rodent malaria parasites, such as *P. yoelii* or *P. berghei*, as a challenge model [8], [9], [10], [11], [12], [13]. As described in the Introduction, a small number of candidate human malaria vaccines based on viral vectors have entered human clinical trials in recent years. Ad5 vectors expressing PfCS and PfAMA-1 were shown to elicit a significant level of CD8+ T-cell response, but a modest degree of protection, with a correlation reported between the level of the CD8+ T-cell response and the degree of protection [22], [23]. The reason why only the modest degree of the protective immunity could be induced by Ad5 vector-based vaccines was, in part, due to the pre-existing anti-Ad5 immunity present in the vaccinees. Nevertheless, this suggests that CD8+ T cells contribute to the protective anti-malaria immune response induced by malaria vaccines. However, as it is impossible to deplete CD8+ T cells in human vaccine recipients, to date, no conclusive evidence confirming a protective role for human CD8+ T cells against malaria has been published.

We recently established a HIS-CD8 mouse model in which AdPfCSP immunization induces an HLA-A∗0201-restricted, PfCSP-specific human cytotoxic CD8+ T-cell response [27]. In the current study, therefore, we sought to determine the role human CD8+ T cells play in the protective anti-malaria immune response using HIS-CD8 mice. We have to note that the number of mice used per group is limited due to the difficulty of expanding HIS-CD8 mice that can possess a high percentage of human lymphocytes in their blood. The difficulty includes the breeding of NSG mice, availability of HLA-A∗0201 matched HSCs, and a long duration (>15 weeks) after HSCs engraftment for human lymphocytes, including human CD8+ T cells, to fully develop and expand in HIS-CD8 mice. Nevertheless, we found that AdPfCSP immunization was able to induce a significant level of both PfCSP-specific human CD8+ T-cell response and protective immune response against challenge with transgenic *P. berghei* sporozoites expressing full-length PfCSP. The level of malaria protection was positively correlated with the level of the PfCSP-specific CD8+ T-cell response. Furthermore, depletion of human CD8+ T cells in AdPfCSP-immunized HIS-CD8 mice almost completely abolished the malaria protection, thus indicating that human CD8+ T cells mediate protective immunity against malaria, most likely against the liver stages of the parasite.

In our previous studies, we showed that hepatocytes in HIS-CD8 mice express HLA-A∗0201 following delivery of the gene via AAV9 and that PfCSP-specific human CD8+ T cells induced by AdPfCSP immunization recognize HLA-A∗0201+ hepatocytes presenting a peptide corresponding to the CD8+ T-cell epitope of PfCSP [27]. In addition, because they were transduced with only the HLA-A∗0201 gene, the HIS-CD8 mice can harbor functional human CD8+ T cells but not functional human CD4+ T or B cells [27]. Taken together, our results suggest that HLA-A∗0201-restricted human CD8+ T cells almost solely mediate the protective anti-malaria immune response, with no contribution by humoral or CD4+ T-cell responses. Thus, our data highlight the importance of designing human malaria vaccines that elicit a protective CD8+ T-cell response.

## Funding

This work was supported by a Grand Challenges Exploration grant (OPP1007283 GCE) from the Gates Foundation and by grants from the Mark S. Bertuch AIDS Research Fund.

## Author contributions

M.T., X.L., and J.H. designed the study. X.L. performed the immunology and parasitology experiments. X.L. and J.H. generated the HIS-CD8 mice, and X.L., and J.H. maintained the HIS-CD8 mice. D.S., R.S., and A.C. provided the transgenic parasites, and M.Z. and R.F. maintained the parasites. M.T. and X.L. analyzed the data. M.T., X.L., V.N., and R.S.N. prepared the manuscript.

## Conflict of Interest

The authors do not have any conflict of interest.
